# Characterization of transcription factor response kinetics in parallel

**DOI:** 10.1186/s12896-016-0293-6

**Published:** 2016-08-24

**Authors:** Betul Bilgin, Aritro Nath, Christina Chan, S. Patrick Walton

**Affiliations:** 1 Department of Chemical Engineering and Materials Science, Michigan State University, 428 S. Shaw Lane, Room 3249, Engineering Building, East Lansing, MI 48824-1226 USA; 2 Genetics Program, Michigan State University, East Lansing, MI 48824 USA; 3 Department of Biochemistry and Molecular Biology, Michigan State University, East Lansing, MI 48824 USA

**Keywords:** Transcription factors, Parallel, Palmitic acid treatment, HepG2 cells, Kinetics, MDA-MB-231 cells

## Abstract

**Background:**

Transcription factors (TFs) are effectors of cell signaling pathways that regulate gene expression. TF networks are highly interconnected; one signal can lead to changes in many TF levels, and one TF level can be changed by many different signals. TF regulation is central to normal cell function, with altered TF function being implicated in many disease conditions. Thus, measuring TF levels in parallel, and over time, is crucial for understanding the impact of stimuli on regulatory networks and on diseases.

**Results:**

Here, we report the parallel analysis of temporal TF level changes due to multiple stimuli in distinct cell types. We have analyzed short-term dynamic changes in the levels of nuclear factor kappa-light-chain-enhancer of activated B cells (NF-kB), signal transducer and activator of transcription 3 (Stat3), cAMP response element-binding protein (CREB), glucocorticoid receptor (GR), and TATA binding protein (TBP), in breast and liver cancer cells after tumor necrosis factor-alpha (TNF-α) and palmitic acid (PA) exposure. In response to both stimuli, NF-kB and CREB levels were increased, Stat3 decreased, and TBP was constant. GR levels were unchanged in response to TNF-α stimulation and increased in response to PA treatment.

**Conclusions:**

Our results show significant overlap in signaling initiated by TNF-α and by PA, with the exception that the events leading to PA-mediated cytotoxicity likely also include induction of GR signaling. These results further illuminate the dynamics of TF responses to cytokine and fatty acid exposure, while concomitantly demonstrating the utility of parallel TF measurement approaches in the analysis of biological phenomena.

**Electronic supplementary material:**

The online version of this article (doi:10.1186/s12896-016-0293-6) contains supplementary material, which is available to authorized users.

## Background

Proper cellular responses to their microenvironments are crucial for cell and tissue homeostasis. Abnormal cellular signaling can lead to diseases such as cancer and diabetes [1–5]. Improved understanding of signaling has led to improvements in disease diagnosis, the understanding of developmental processes, and the engineering of artificial tissues [6–8]. In fact, recent advances in cell signaling pathway analysis have led to the advent of a new field of study, systems biology [9]. Systems biology relies on vast amounts of data that can be generated from the various “omics” techniques. These data represent the results of cell signal transduction and would be complemented by parallel measurements of the levels of transcription factors, the upstream mediators of cellular signaling.

Transcription factors (TFs) are responsible for altering the cell state in response to stimuli by changing the transcription rates of their targeted genes. They interact with specific sites on genomic DNA, often recruiting other co-factors to the location, resulting in activation or repression of the corresponding genes. Analysis of cell signaling responses to stimuli is complicated by the fact that TFs generally control several genes, most genes are controlled by multiple TFs, and any given stimulus can result in the activation of multiple TFs [10–12]. Moreover, TF levels must change in response to a stimulus but, in most cases, must then return to baseline levels to avoid long-term perturbation of cellular function [13, 14]. The TFs that control genes involved in the rapid response to stimuli are often present in the cytoplasm in an inactive state, translocated to the nucleus after activation, and then destroyed or trafficked out of the nucleus to halt their function [15–18]. Thus, tracking TF activities requires analysis across many dimensions, including activation status, subcellular location, and time. With an estimated 2000 TFs in humans, profiling the levels of active TFs is a dynamic and complex task.

To address this challenge, we and others are developing quantitative, sensitive, parallel techniques for measurement of TF levels [19–22]. We have previously demonstrated parallel measurements of TFs in extracts from breast cancer cells using our magnetic bead-based assay [23]. Our approach aims to simplify measuring TFs in parallel, by detecting TF-bound dsDNA, rather than the TF itself. In this way, a challenging task, parallel protein detection, is reformulated into a relatively straightforward task, parallel nucleic acid detection. In this study, we have expanded the number of TFs analyzed and applied our method to the kinetic analysis of cellular responses to multiple stimuli, specifically to cytokines (tumor necrosis factor-α, TNF-α) and saturated fatty acids (palmitic acid, PA).

## Methods

### DNA probe design and radiolabeling

Detailed design information and probe sequences are listed in the (Additional file 1: Table S1). ssDNAs were purchased from Integrated DNA Technologies (Coralville, IA). Probes were generated by hybridization of equimolar amounts of complementary ssDNAs in 1× STE buffer (10 mM Tris, 100 mM NaCl, and 1 mM EDTA), heating to 95 °C for 5 min, followed by incubation at room temperature for 1 h. After hybridization, dsDNA probes were 5′-radiolabeled with 10 pmoles of [γ-^33^P] ATP using T4 polynucleotide kinase (New England Biolabs, Ipswich, MA). Radiolabeled DNA probes were purified from unincorporated label with G-25 Sephadex columns (Roche Applied Science; Indianapolis, IN).

### Cell culture

Our experiments were conducted with two different cell lines, HepG2 (human hepatocellular carcinoma, obtained from ATCC) and MDA-MB-231 (human breast adenocarcinoma, generously provided by Kathleen Gallo, Department of Physiology, Michigan State University). HepG2 cells were cultured in Dulbecco’s Modified Eagle Medium (Life Technologies; Grand Island, NY) supplemented with 10 % fetal bovine serum (Life Technologies; Grand Island, NY), 100 μg/mL streptomycin (Life Technologies; Grand Island, NY) and 100 U/mL penicillin (Life Technologies; Grand Island, NY) in a humidified incubator at 37 °C and 5 % CO_2_. For TNF-α treatments, cells were grown to 90 % confluence in 6-well plates and then treated with 50 ng/ml recombinant human TNF-α (R&D Systems; Minneapolis, MN) for periods of 0.5, 1, 2, 4 and 24 h. Control cells received fresh media without TNF-α. For palmitic acid treatments, cells were grown to 90 % confluence in 6-well plates and then treated with media containing 0.7 mM palmitic acid (Sigma; St. Louis, MO) complexed with 2 % (w/v) fatty acid free BSA (US Biologicals; Salem, MA) for 0.5, 1, 2, 4, 16, and 24 h. Control cells were grown in media containing 2 % BSA for the same time periods.

MDA-MB-231 cells were cultured in Dulbecco’s Modified Eagle Medium (Life Technologies; Grand Island, NY) with 10 % fetal bovine serum, 2 mM glutamine (Life Technologies; Grand Island, NY), 100 μg/mL streptomycin, and 100 U/mL penicillin. Cells were maintained in a humidified incubator at 37 °C and 10 % CO_2_ as described [24]. For TNF-α treatments, cells were grown to 90 % confluence in 6-well plates and then treated with 50 ng/ml recombinant human TNF-α for periods of 0.5, 1, 2, 4 and 24 h. Control cells received fresh media without TNF-α.

### Nuclear extraction

For HepG2 cells, nuclear extraction was performed as described [25]. Briefly, after washing the cells with PBS, cells were resuspended and allowed to swell in five times the packed cell volume (PCV) of ice cold buffer (10 mM HEPES (pH = 7.9) (Sigma; St. Louis, MO), 10 mM KCl, 0.1 mM EDTA, freshly added protease inhibitors (complete mini EDTA free cocktail tablets, Roche), and phosphatase inhibitors (phosphatase inhibitor cocktail, Sigma)) for 15 min. After adding 10 % NP-40 solution to a final concentration of 0.5 % (v/v), cells were vortexed for 20 s, and nuclear pellets were collected by centrifugation at 13,000 *g* for 1 min at 4 °C. Nuclear pellets were washed three times with buffer (same as above) and resuspended in 1×PCV of ice cold buffer (20 mM HEPES pH 7.9, 0.4 M NaCl, 1 mM EDTA, freshly added protease inhibitors (complete mini EDTA free cocktail tablets, Roche), and phosphatase inhibitors (phosphatase inhibitor cocktail, Sigma)). Pellets were shaken for 15 min, and nuclear extracts were obtained by centrifugation at 13,000 *g* for 15 min at 4 °C. The total protein concentration in all extracts was measured by BCA Protein Assay kit (Thermo Scientific Pierce; Rockford, IL).

For MDA-MB-231 cells, nuclear extraction was performed as described [26]. Briefly, after washing the cells with PBS, the cells were trypsinized and allowed to swell in buffer (10 mM HEPES (pH = 8.0), 1.5 mM MgCl_2_, 10 mM KCl, protease inhibitor (complete mini EDTA free cocktail tablets, Roche)) on ice for 15 min. The cells were then lysed with 15 strokes of a 25-gauge, 5/8 inch needle, and the nuclear pellets were collected by centrifugation at 12,000 *g* for 15 min. Nuclear pellets were resuspended and incubated in a second buffer (20 mM HEPES (pH = 8.0), 1.5 mM MgCl2, 25 % glycerol, 420 mM NaCl, 0.2 mM EDTA (pH = 8.0), protease inhibitor) on ice for 30 min. After incubation, nuclear extracts (supernatants) were obtained following centrifugation at 12,000 *g* for 5 min.

It should be noted that complete separation of nuclear and cytoplasmic extracts is crucial for accurate TF analyses. High fidelity separations preclude latent TFs in the cytoplasmic fraction from giving a false positive signal for the nuclear extracts [27]. To assess nuclear/cytoplasmic separation, we conducted western blots for TBP (nuclear protein) and GAPDH (principally cytoplasmic protein) (Additional file 2: Figure S1).

### TF biotinylation

Nuclear extracts were chemically biotinylated by EZ-Link-Iodoacetyl-PEG_2_-biotin (Thermo Scientific Pierce; Rockford, IL), according to the manufacturer’s instructions. Nuclear extracts were mixed with EZ-Link-Iodoacetyl-PEG_2_-biotin in reaction buffer (50 mM Tris–HCl, 5 mM EDTA, pH 8.0) at RT for 90 min. Biotinylated TFs were purified with G-50 Sephadex columns (Roche Applied Science; Indianapolis, IN). Sephadex columns were washed three times with PBS prior to use.

### Magnetic bead-based TF quantification

TFs were measured according to our established technique [23]. Biotin labeled TFs were immobilized on streptavidin-coated magnetic beads (Dynal/Invitrogen; Oslo, Norway) by incubation at RT for 20 min in 1× PBS. After applying a magnet, the supernatant was removed and the TF-bound beads recovered. Following three washes, the TF-loaded beads were mixed with dsDNA probes in binding buffer for 20 min at RT. The supernatant was collected by applying the magnet again, and the beads were washed twice with washing buffer (0.02 % Tween 20 in water) with the supernatants collected after each wash step.

#### Scintillation counting

For single TF measurements, the technique was performed using one radiolabeled dsDNA probe while all other probes were unlabeled. After binding to the TF-loaded beads and washes to remove non-specifically bound probes, the beads were resuspended in 50 μl of water and then mixed with 10 ml Safety Solve High Flash Point Scintillation Cocktail (Research Products International Corporation; Mount Prospect, IL). The signal from all fractions (supernatant, wash 1, wash 2, and beads) was measured, and the percentage of the signal retained on the beads was calculated. All signals were normalized to control to account for decay in the radiolabel on the probe.

#### Electrophoresis readout

For parallel TF measurements, dsDNA probes were bound to the TF-loaded beads. After washes, the beads were resuspended in 1×TBE and heated at 95 °C for 15 min to elute the retained DNA. A magnet was applied and the supernatant collected. Eluted DNA probes (1 μl of the 25 μl) were mixed with universal primers (300 nM) and amplified for 20 cycles with Taq DNA Polymerase (New England Biolabs; Ipswich, MA) in 50 μl reactions. The PCR program was: 95 °C for 30 s (melting), 61 °C for 30 s (annealing), and 72 °C for 10 s (extension). 12 μl of PCR product was mixed with 4 μl of gel loading buffer, and 14 μl was loaded onto native 4–12 % TBE gels. Gels were run at 300 V for 30 min on ice, stained with SYBR Gold (Invitrogen; Carlsbad, CA), and visualized in a ChemiDoc XRS System (Bio-Rad; Hercules, CA). Band intensities were quantified by QuantityOne software. For normalization, each signal was normalized to an internal standard included in each PCR reaction. The internal standard included the universal primer sites but did not contain a TF binding site. This accounted for run-to-run differences in PCR efficiency and gel exposure. After signals were normalized to the internal standard, the ratio of treated to control was calculated. We have previously validated the quantitative binding and release of probes from the TFs bound to the beads and all other steps prior to PCR [23].

### TF measurements by EMSA

Biotinylated TFs were mixed with dsDNA probes (one probe radiolabeled, the rest unlabeled) in a 20 μl reaction volume of binding buffer (10 mM Tris–HCl, 1 mM MgCl_2_, 0.5 mM EDTA, 0.5 mM DTT, 50 mM NaCl, 1 mM CaCl_2_, 0.2 mM KCl, 10 mM ZnCl_2_, 4 % glycerol, 20 mM acetic acid, 0.025 μg/μL poly (dI-dC)) for 30 min at RT. Biotinylated TF concentrations were measured by Bradford assay with 2 μg of protein loaded per assay. 15 μl of the binding reaction were mixed with 5 μl of gel loading buffer, and 18 μl was loaded onto native 4–12 % TBE gels. Gels were run at 300 V for 30 min on ice, dried, and detected by phosphorimaging using the Storm 860 (GE Healthcare; Pittsburgh, PA). Band intensities were quantified by QuantityOne software. A representative image is provided in the (Additional file 3: Figure S2).

### Statistical analyses

All experiments were performed at least 3 times. For gel images, representative results are shown. All error bars show the mean +/− SD value of experiments performed. Two-way student *t*-test was used to evaluate statistical significance of values compared to control samples. The *t*-test was conducted as follows: a pooled variance was calculated for all time points for a given TF to account for the distribution of variances across the different samples. A t-statistic was calculated from the degrees of freedom for each TF and was used to build a 95 % confidence interval about the mean for each TF at each time point. Significance relative to normalized controls was determined by whether the value of 1 did (not significant) or did not (significant) fall within the 95 % confidence interval. This analysis was developed in consultation MSU’s Center for Statistical Training and Consulting (CSTAT).

## Results and discussion

Having previously demonstrated applicability of our approach to parallel measurement of TFs [23], we sought to apply the technique to furthering our understanding of biological signaling kinetics while also demonstrating use of the technique for measuring a broader array of TFs in parallel. We chose to examine cytokine stimulation and fatty-acid exposure of cells as stimuli and designed our panel of TFs according to our expectations about which pathways could be activated by these stimuli. In addition, we tested our technique using extracts from two unique cell types to demonstrate the applicability of the method to different cell systems and to examine the commonalities and differences in response among the systems and stimuli.

### TF measurements in MDA-MB-231 breast cancer cells stimulated with TNF-α

**Fig. 1:**
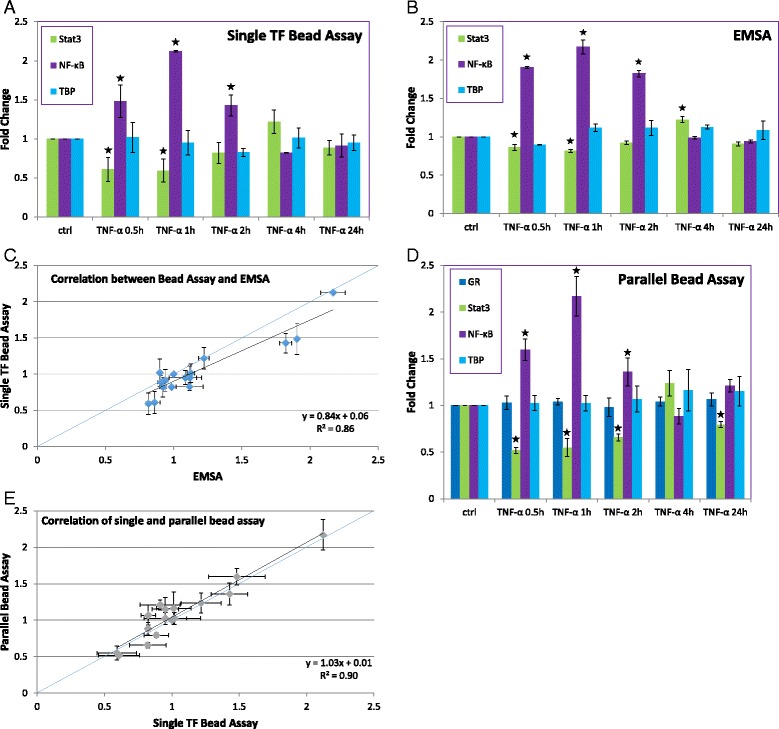
Quantification of TF levels in nuclear extracts of MDA-MB-231 cells after TNF-α stimulation. **a** Single TF detection by bead assay. The percentage of radiolabeled TF probe remaining on the beads (relative to signal that did not bind or was washed from the beads) was calculated. Fold changes relative to control are shown. (*n* = 3, * indicates *p* < 0.05). **b** Single TF detection by EMSA. The fractions of bound and unbound DNA probe were quantified, and the fraction of bound signal was calculated relative to the total signal from the lane. Fold changes relative to control are shown. (*n* = 3, * indicates *p* < 0.05). **c** Correlation between detection by bead assay and EMSA. The 1:1 line (*blue*) is included for reference. **d** Parallel TF detection by bead assay. Signals were normalized with respect to an internal standard and then the ratio with respect to control was calculated. (*n* = 3, * indicates *p* < 0.05). **e** Correlation of single and parallel bead assay measurements. The 1:1 line (*blue*) is included for reference

We first examined the changes in nuclear TF levels associated with TNF-α stimulation of MDA-MB-231 breast cancer cells. These experiments were intended to serve as an initial validation of our larger set of TF measurements. For these experiments, the levels of four different TFs were measured in parallel with respect to treatment time (Fig. 1). These TFs were nuclear factor kappa-light-chain-enhancer of activated B cells (NF-kB), signal transducer and activator of transcription 3 (Stat3), glucocorticoid receptor (GR), and TATA binding protein (TBP). For measurements of individual TFs (in which we only tested NF-kB, Stat3, and TBP), our results showed an increase in nuclear NF-kB levels after TNF-α stimulation, with levels peaking at 1 h after stimulation (Fig. 1a). NF-kB levels returned to baseline after approximately 4 h, remaining at or near baseline levels for the remainder of the experiment. On the other hand, Stat3 levels decreased after 0.5 h, returning to baseline levels after 2 h. Our expected control TF, TBP, showed no significant change over the course of the experiment. To confirm our results, we performed electrophoretic mobility shift assays (EMSAs) on each of the samples. Agreement was seen between the EMSA (Fig. 1b) and measurements by our method (Fig. 1c) in the data trends (increases and decreases in TF levels) and generally in the magnitude of the changes, as well.

Our objective is to expand the technique to detection of hundreds of TFs in parallel with a high throughput technique such as parallel sequencing. For our current scale of 4 TFs in parallel, a PCR-based readout was applied as a proxy for such readouts. Each dsDNA probe was designed to include universal sense and antisense primer sequences and to yield a PCR product of unique length following amplification. To accomplish this, we included different numbers of binding sequence repeats for some TFs (Additional file 1: Table S1). In our experiments, eluted DNAs from magnetic beads were PCR amplified with universal primers for 20 cycles, to achieve a semi-quantitative PCR readout. For all TFs, our single protein measurements with EMSA and scintillation counting correlated well with the levels measured in parallel (Fig. 1d and e). In all detection techniques, NF-kB levels were increased within 0.5 h and returned to baseline after 4 h. In contrast, Stat3 levels decreased and recovered over the same time period. GR levels were unchanged with TNF-α treatment. This was our expectation based upon prior work in muscle cells [28]. The changes in NF-kB and STAT levels agree with previously published reports examining the responses of these TFs to TNF-α stimulation [29, 30], though these were measured at a single timepoint. Our kinetic data demonstrate that TF levels in the nucleus can change rapidly both in the initiation of the response and in the return to baseline levels.

### TF measurements in HepG2 cells stimulated with TNF-α

**Fig. 2:**
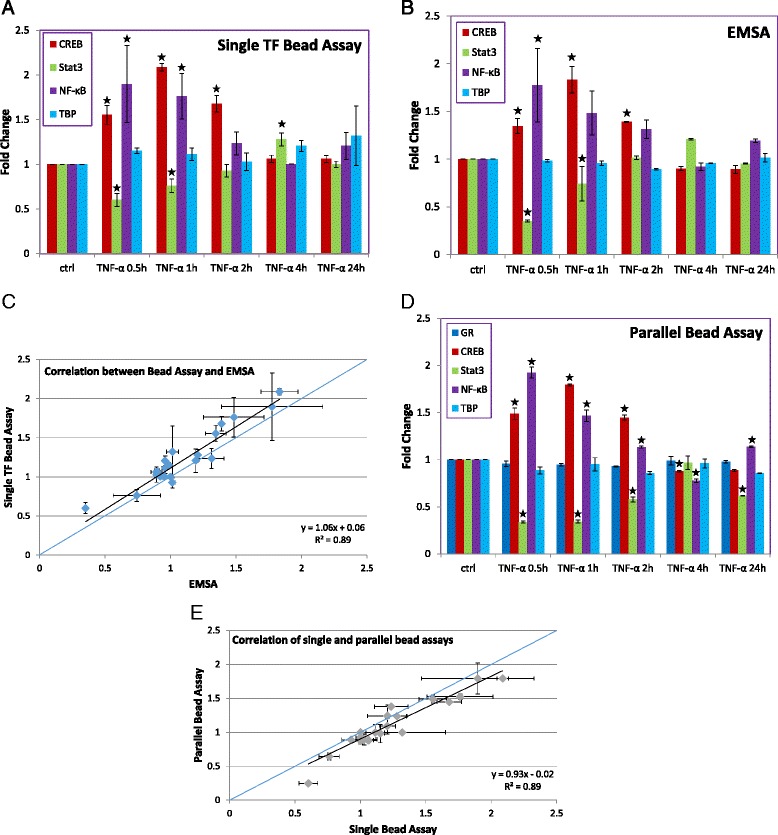
Quantification of TF levels in nuclear extracts of HepG2 cells after TNF-α stimulation. **a** Single TF detection by bead assay. The percentage of radiolabeled TF probe remaining on the beads (relative to signal that did not bind or was washed from the beads) was calculated. Fold changes relative to control are shown. (*n* = 3, * indicates *p* < 0.05). **b** Single TF detection by EMSA. The fractions of bound and unbound DNA probe were quantified, and the fraction of bound signal was calculated relative to the total signal from the lane. Fold changes relative to control are shown. (*n* = 3, * indicates *p* < 0.05). **c** Correlation between detection by bead assay and EMSA. The 1:1 line (*blue*) is included for reference. **d** Parallel TF detection by bead assay. Signals were normalized with respect to an internal standard and then the ratio with respect to control was calculated. (*n* = 3, * indicates *p* < 0.05). **e** Correlation of single and parallel bead assay measurements. The 1:1 line (*blue*) is included for reference

To test the feasibility of our assay with different cell types, we measured TF levels in HepG2 cells. cAMP response element-binding protein (CREB) was included in this set of measurements. We and other have previously studied the effects of TNF-α on these TFs [31, 32]. As with the breast cancer cell experiments, individual TF levels were detected with scintillation counting and EMSA (Fig. 2a, b), while multiple TFs were detected by our parallel PCR readout (Fig. 2d). Results from the three different techniques correlated well, showing NF-kB and CREB levels increasing, with CREB peaking and returning to baseline later than NF-kB (Fig. 2c and e). Over a similar timeframe, Stat3 levels decreased and also returned to baseline. After 4 h, all measured TF levels returned to control levels. TBP and GR levels were unchanged during the measurement period. These results align with those from the breast cancer cell experiments (Fig. 1).

In hepatocytes, TNF-α has been shown to initiate several different responses, including cytotoxicity [33]. However, hepatocytes are capable of resisting the cytotoxic effects of TNF-α by activating NF-kB [34]. Additionally, recent studies have suggested that TNF-α signaling can activate the IKK/JNK pathway [32, 35], which, in turn, enhances CREB activity [36, 37]. Both NF-kB and CREB activate transcription of anti-apoptotic and cell proliferation genes [20, 38]. In line with this possible defensive response, we found rapid activation of both TFs by TNF-α.

The reduced nuclear levels of Stat3 may suggest a rapid export/degradation of nuclear Stat3 or a reduction in the rate of translocation from the cytoplasm to the nucleus. It is known that Stat3 localized to the cytoplasm may interact with protein kinase R (PKR) and inhibit its phosphorylation activity [39]. PKR inhibits translation initiation and induces apoptosis via the FADD-dependent Caspase 8 pathway [40]. Thus, lower levels of Stat3 in the nucleus, if accompanied by concomitant increases in cytoplasmic Stat3, may suggest a possible anti-apoptotic effect mediated by PKR repression.

For additional validation of our measured responses, we also compared the TF measurements to western blotting of each TF, with qualitative but not quantitative agreement (Additional file 4: Figure S3). Western blotting is a technique that measures the total quantity of TF protein, while our approach and EMSA specifically measure the quantity of active TF (as defined by its ability to bind its dsDNA recognition sequence). Since western blotting consistently showed elevated signals relative to the other approaches, this suggests that either TFs are being inactivated during sample preparation, which seems unlikely given the activity of the remaining proteins, or that a large fraction (~50 %) of the TF molecules that are present in the nucleus are inactive prior to destruction or trafficking out of the nucleus [41]. Our data also demonstrate how important kinetic measurements of TF levels can be to understanding biological processes. For our TFs, measurements at 24 h alone would have shown no response, hiding the early events in response to the stimulus that likely contribute to downstream changes in cellular function.

### TF measurements in HepG2 cells stimulated with palmitic acid

**Fig. 3:**
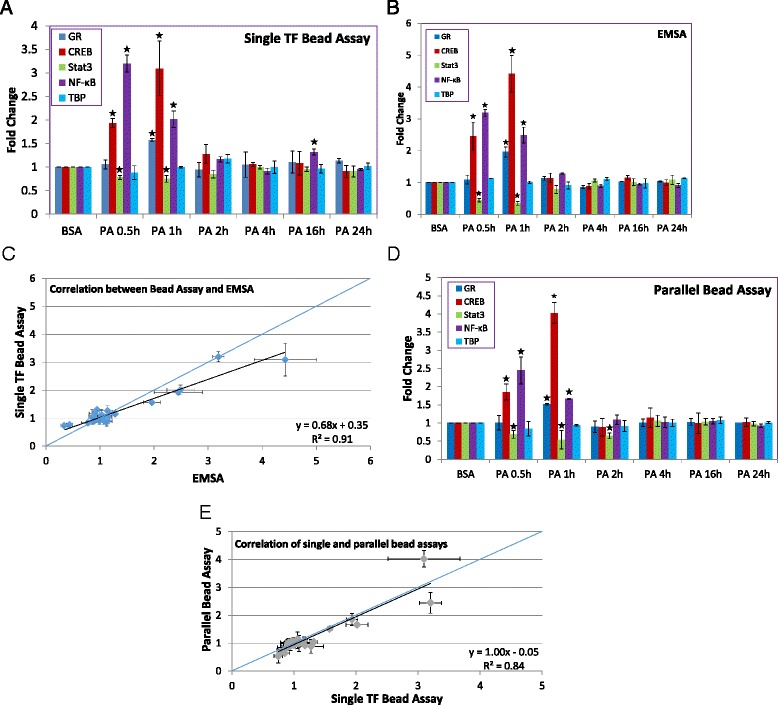
Quantification of TF levels in nuclear extracts of HepG2 cells after palmitic acid treatment. **a** Single TF detection by bead assay. The percentage of radiolabeled TF probe remaining on the beads (relative to signal that did not bind or was washed from the beads) was calculated. Fold changes relative to control are shown. (*n* = 3, * indicates *p* < 0.05). **b** Single TF detection by EMSA. The fractions of bound and unbound DNA probe were quantified, and the fraction of bound signal was calculated relative to the total signal from the lane. Fold changes relative to control are shown. (*n* = 3, * indicates *p* < 0.05). **c** Correlation between detection by bead assay and EMSA. The 1:1 line (blue) is included for reference. **d** Parallel TF detection by bead assay. Signals were normalized with respect to an internal standard and then the ratio with respect to control was calculated. (*n* = 3, * indicates *p* < 0.05). **e** Correlation of single and parallel bead assay measurements. The 1:1 line (blue) is included for reference

In recent years, we and others have focused on the cytotoxic effects of FFAs on hepatocytes [42, 43]. However, the signaling pathways associated with palmitic acid (PA) exposure are not well-established. Thus, we wanted to address which TFs of our analytical set are involved in the cellular response to PA exposure. Prior work had identified a role for NF-kB and CREB [44]. Our particular interest in GR is two-fold. First, GR was shown to increase activated CREB levels [45], and CREB can act with GR to regulate genes where their binding sites are within 90 bp of each other [46]. As above, TF expression levels were measured by scintillation counting or EMSA for individual TFs, or PCR for TFs in parallel (Fig. 3) (A representative image of the parallel PCR readout is shown in the Additional file 5: Figure S4). Our data consistently show that NF-kB activity increased and reached a maximum at 30 min, then returned to baseline by 2 h, with a similar pattern observed with CREB. As with our TNF-α studies, nuclear Stat3 levels decreased by 30 min and returned to basal levels by 2 h. Importantly, GR levels were increased by PA treatment, peaking at 1 h. TBP levels were unchanged as expected.

These data show that HepG2 cells respond to PA treatment by dynamic changes in the trafficking of these TFs into or out of the nucleus. Of the TFs we measured, NF-kB was most rapidly activated, followed soon after by CREB and GR. The increased activities of NF-kB and CREB in response to elevated free fatty acids is in agreement with prior studies [44]. Given that elevated levels of PA are cytotoxic to HepG2 cells [47], this response suggests that NF-kB and CREB are activated to initiate an anti-apoptotic response to this stimulus, as in the TNF-α experiments.

### Comparison of TNF-α and PA responses

The NF-kB, CREB and Stat3 responses of HepG2 cells to palmitic acid and TNF-α are similar, indicating that similar pathways are activated by each stimulus. It has already been shown that circulating and liver levels of TNF-α are elevated in non-alcoholic fatty liver disease (NAFLD) [48, 49]. Additionally, free fatty acids have been shown to elevate TNF-α expression [50]. It is unclear if TNF-α signaling is associated with the response to PA that we have measured, given the short time scales over which we detect significant changes in TF levels. It may be that the similarity in the responses is related to existing crosstalk between the two pathways.

Intriguingly, GR responds differently between the TNF-α and PA stimulated experiments. Currently, no data support a role of GR in mediating PA-induced cytotoxicity. However, it has been shown that elevated levels of glucocorticoids may be associated with pathogenesis of NAFLD [51]. Also, in previous studies, GR has been shown to significantly enhance activated CREB levels [44], with both being increased in our results. GR and CREB are known to synergistically activate expression of certain genes, e.g., phosphoenolpyruvate carboxykinase (PEPCK), somatostatin, and even GR itself [52, 53]. Importantly, activation of PEPCK by palmitic acid was observed with HepG2 cells previously [54]. It has also been shown that there is crosstalk between GR and CREB in regulating neuronal genes [55]. Taken together, our results and the literature suggest that genes regulated by GR (and perhaps particularly those co-regulated by CREB and GR) might be important in the cellular response to saturated fatty acid (palmitic acid) exposure. Stat3 may also respond differently to TNF-α and PA. While the early downregulation of Stat3 levels is consistent between the stimuli, in some of the TNF-α analyses, Stat3 levels at 4 h are slightly elevated (e.g., Figs. 1b, 2a). This may be a result of a feedback mechanism to restore Stat3 to basal levels. Additional timepoints between 4 h and 24 h may have clarified whether the observed increase in Stat3 was biologically meaningful.

### Future development and experiments

In this work, we have shown that our assay can be applied to measure TF levels in parallel over time and is useful for the analysis of TFs involved in cytokine and fatty acid treatments of cultured cells. For both of our model systems, we used cancer cell lines with which we have considerable experience. We acknowledge that the responses we detected may not be universal across all human cell types but are nonetheless useful in demonstrating the ability of our method to detect changes in TF levels. We believe that our assay can be used to profile other TF pathways and to study early cellular responses.

We recognize that we only measured a limited number of TFs in parallel and only over a 24 h time period. Subsequent work and redesign of the technique will focus on inclusion of late-acting TFs, whose expression level changes would be expected after 8 h. Complementing our analytical approach with chromatin immunoprecipitation (ChIP), either for individual TFs or in parallel, would further our understanding of the downstream signaling that results from the nuclear TFs we measure. This combination of early-acting and late-acting TFs would considerably enhance the information provided by the technique in analyzing biological systems. Combined with other parallel analytical techniques, our approach would contribute to a more complete picture of TF regulation and signaling in response to a variety of stimuli.

## Conclusions

We have dynamically measured TF levels in response to cytokine and palmitic acid treatments using our previously developed assay. With our assay, NF-kB, CREB, Stat3, GR, and TBP levels were successfully analyzed over 24 h. Our assay can be further improved for profiling larger sets of TFs and could contribute to explorations of cellular regulatory mechanisms, TF pathway mapping, and modeling of dynamic signaling networks.

## Additional files

Additional file 1: Table S1. Sequences and designs of DNA probes and PCR primers. Complementary sequences of universal primers to DNA probes are underlined. Repeats of TF binding sites are shown in design with the repeat of the same color. (DOCX 523 kb) Additional file 2: Figure S1. Representative western blots showing separation of nuclear (lanes 1–6 from left) and cytoplasmic (lanes 7–12) fractions. Detection was for either TBP (top) or GAPDH (bottom). (DOCX 677 kb) Additional file 3: Figure S2. Representative image of EMSA detection of TF binding. Replicate experiments for detection of CREB in TNF-α treated (50 ng/ml) HepG2 cells. Lanes 1, 7 – No TNF-α; Lanes 2, 8–0.5 h; Lanes 3, 9–1 h; Lanes 4, 10–2 h; Lanes 5, 11–4 h; Lanes 6, 12–24 h. (DOCX 1 mb) Additional file 4: Figure S3. Western blot analysis of NF-kB levels in TNF-α treated HepG2 cells. Representative western blot image and quantification of NF-kB levels from multiple western blots. (DOCX 606 kb) Additional file 5: Figure S4. Parallel TF detection by bead assay. Representative image of a parallel PCR readout (PA treated HepG2 samples). (DOCX 580 kb)

## Abbreviations

BSA: Bovine serum albumin
CREB: cAMP response element-binding protein
CSTAT: Center for statistical training and consulting
DTT: Dithiothreitol
EDTA: Ethylenediaminetetraacetic acid
EMSA: Electrophoretic mobility shift assay
FFA: Free fatty acid
GR: Glucocorticoid receptor
HEPES: 4-(2-hydroxyethyl)-1-piperazineethanesulfonic acid
HepG2: Human hepatocellular carcinoma
MUCI: Michigan universities commercialization initiative
NAFLD: Non-alcoholic fatty liver disease
NF-kB: Nuclear factor kappa-light-chain-enhancer of activated B cells
PA: Palmitic acid
PBS: Phosphate-buffered saline
PCR: Polymerase chain reaction
PCV: Packed cell volume
PEPCK: Phosphoenolpyruvate carboxykinase
PKR: Protein kinase R
SD: Standard Deviation
Stat3: Signal transducer and activator of transcription 3
STE: Sodium Chloride-Tris-EDTA
TBE: Tris-Borate-EDTA
TBP: TATA binding protein
TF: Transcription factor
TNF-α: Tumor necrosis factor-alpha
