# Supplementary material for: Characterization of transcription factor response kinetics in parallel
Source: BMC Biotechnol. 2016 Aug 24;16(1):62. doi: 10.1186/s12896-016-0293-6 (PMC4997724; doi:10.1186/s12896-016-0293-6)
Supplement: Additional file 1: Table S1. — Sequences and designs of DNA probes and PCR primers. Complementary sequences of universal primers to DNA probes are underlined. Repeats of TF binding sites are shown in design with the repeat of the same color. (DOCX 523 kb) [file 12896_2016_293_MOESM1_ESM.docx]

**Table S1**

Sequences and designs of DNA probes and PCR primers. Complementary sequences of universal primers to DNA probes are underlined. Repeats of TF binding sites are shown in design with the repeat of the same color.


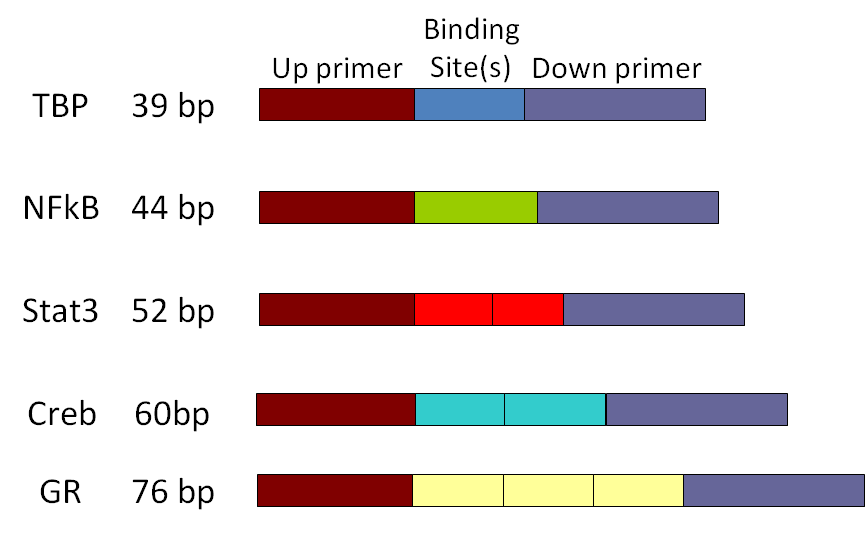


| Transcription Factor | Probe name | Probe sequence |
| --- | --- | --- |
| NF-κB | NF-κB oligo | 5’- GATGTCCACGAGGTCTCTGGAAAGTCCCTACGCTGCAGGTCGAC-3’ |
|  | NF-κB complement | 5’-GTCGACCTGCAGCGTAGGGACTTTCCAGAGACCTCGTGGACATC-3’ |
| CREB | CREB oligo | 5’-GATGTCCACGAGGTCTCTGACGTCAGCGAGTGACGTCAGCGAGCTACGCTGCAGGTCGAC-3’ |
|  | CREB complement | 5’-GTCGACCTGCAGCGTAGCTCGCTGACGTCACTCGCTGACGTCAGAGACCTCGTGGACATC-3’ |
| TBP | TBP oligo | 5’-GATGTCCACGAGGTCTCTTATAATACGCTGCAGGTCGAC-3’ |
|  | TBP complement | 5’-GTCGACCTGCAGCGTATTATAAGAGACCTCGTGGACATC-3’ |
| GR | GR oligo | 5’-GATGTCCACGAGGTCTCTGTACACTGTGTTCTGTACACTGTGTTCTGTACACTGTGTTCTTACGCTGCAGGTCGAC-3’ |
|  | GR complement | 5’-GTCGACCTGCAGCGTAAGAACACAGTGTACAGAACACAGTGTACAGAACACAGTGTACAGAGACCTCGTGGACATC-3’ |
| Stat3 | Stat3 oligo | 5’-GATGTCCACGAGGTCTCTTTCCGGGAATTCCGGGAATACGCTGCAGGTCGAC-3’ |
|  | Stat3 complement | 5’- GTCGACCTGCAGCGTATTCCCGGAATTCCCGGAAAGAGACCTCGTGGACATC-3’ |
| Internal  Standard | Forward primer | 5’-GATGTCCACGAGGTCTCTTACGCTGCAGGTCGAC-3’ |
|  | Reverse primer | 5’-GTCGACCTGCAGCGTAAGAGACCTCGTGGACATC-3’ |
| Universal  Primers | Forward | 5’-GATGTCCACGAGGTCTCT-3’ |
|  | Reverse | 5’-GTCGACCTGCAGCGTA3’ |
