# Supplementary material for: Characterization of transcription factor response kinetics in parallel
Source: BMC Biotechnol. 2016 Aug 24;16(1):62. doi: 10.1186/s12896-016-0293-6 (PMC4997724; doi:10.1186/s12896-016-0293-6)

**FIGURE S1**

Representative western blots showing separation of nuclear (lanes 1-6 from left) and cytoplasmic (lanes 7-12) fractions. Detection was for either TBP (top) or GAPDH (bottom).


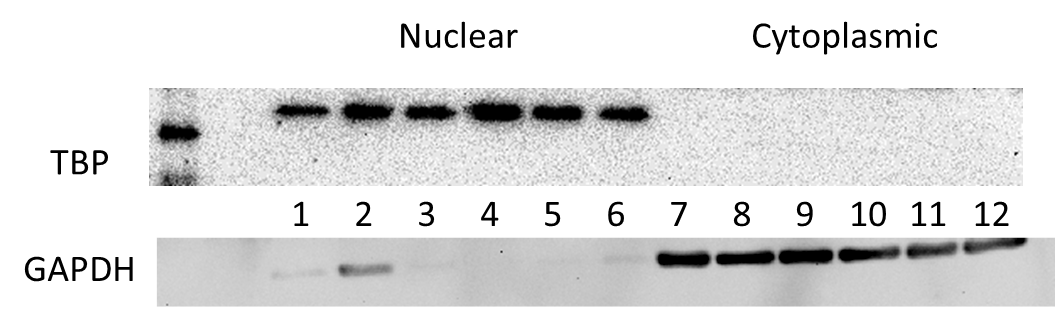

Supplement: Additional file 2: Figure S1. — Representative western blots showing separation of nuclear (lanes 1–6 from left) and cytoplasmic (lanes 7–12) fractions. Detection was for either TBP (top) or GAPDH (bottom). (DOCX 677 kb) [file 12896_2016_293_MOESM2_ESM.docx]
