# Supplementary material for: Characterization of transcription factor response kinetics in parallel
Source: BMC Biotechnol. 2016 Aug 24;16(1):62. doi: 10.1186/s12896-016-0293-6 (PMC4997724; doi:10.1186/s12896-016-0293-6)

**FIGURE S2**

Representative image of EMSA detection of TF binding. Replicate experiments for detection of CREB in TNF-α treated (50 ng/ml) HepG2 cells. Lanes 1, 7 – No TNF-α; Lanes 2, 8 – 0.5 h; Lanes 3, 9 – 1 h ; Lanes 4, 10 – 2 h; Lanes 5, 11 – 4 h; Lanes 6, 12 – 24 h.


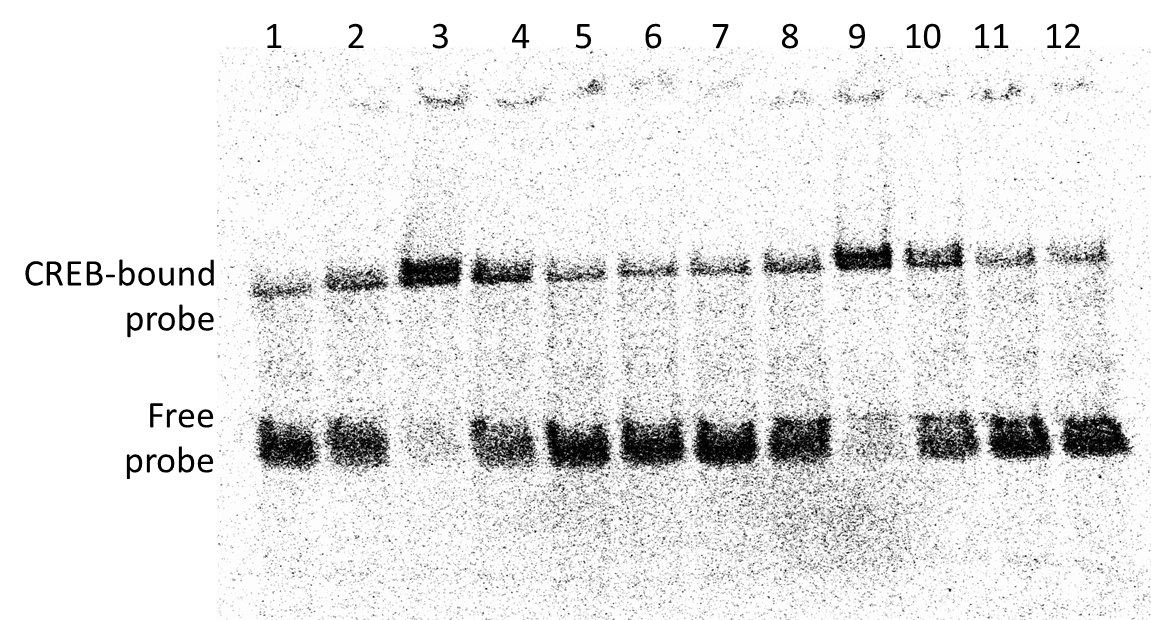

Supplement: Additional file 3: Figure S2. — Representative image of EMSA detection of TF binding. Replicate experiments for detection of CREB in TNF-α treated (50 ng/ml) HepG2 cells. Lanes 1, 7 – No TNF-α; Lanes 2, 8–0.5 h; Lanes 3, 9–1 h; Lanes 4, 10–2 h; Lanes 5, 11–4 h; Lanes 6, 12–24 h. (DOCX 1 mb) [file 12896_2016_293_MOESM3_ESM.docx]
