# Supplementary material for: Characterization of transcription factor response kinetics in parallel
Source: BMC Biotechnol. 2016 Aug 24;16(1):62. doi: 10.1186/s12896-016-0293-6 (PMC4997724; doi:10.1186/s12896-016-0293-6)

**FIGURE S3**

Western blot analysis of NF-κB levels in TNF-α treated HepG2 cells. Representative western blot image and quantification of NF-κB levels from multiple western blots.

0.5h

1h

2h

4h

24h

control

50 ng/ml TNF-alpha


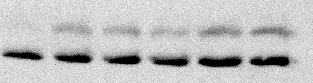


NF-kB

TBP


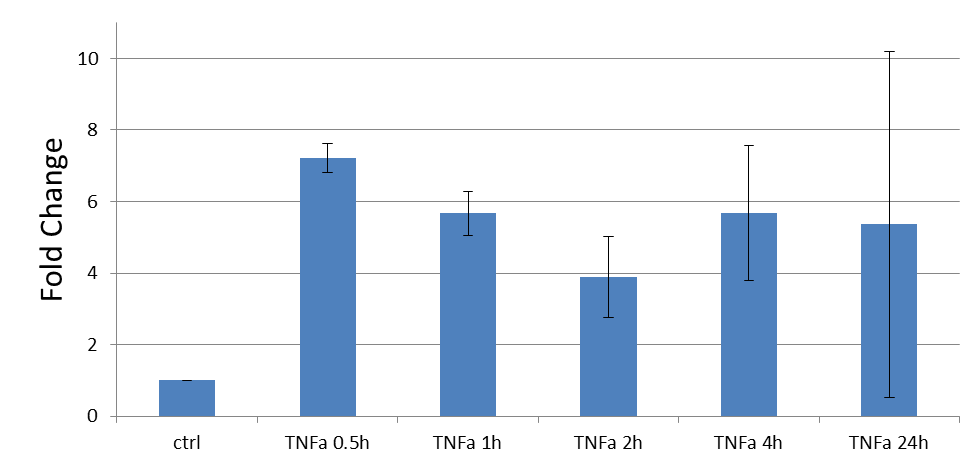

Supplement: Additional file 4: Figure S3. — Western blot analysis of NF-kB levels in TNF-α treated HepG2 cells. Representative western blot image and quantification of NF-kB levels from multiple western blots. (DOCX 606 kb) [file 12896_2016_293_MOESM4_ESM.docx]
