# Supplementary material for: Characterization of transcription factor response kinetics in parallel
Source: BMC Biotechnol. 2016 Aug 24;16(1):62. doi: 10.1186/s12896-016-0293-6 (PMC4997724; doi:10.1186/s12896-016-0293-6)

**FIGURE S4**

Parallel TF detection by bead assay. Representative image of a parallel PCR readout (PA treated HepG2 samples).


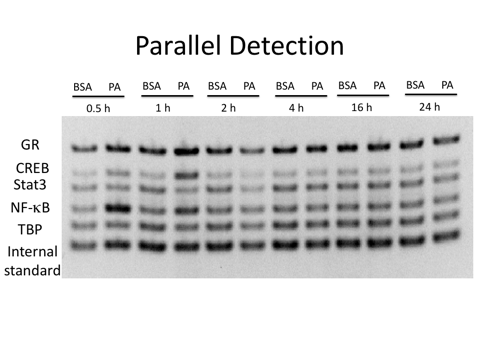

Supplement: Additional file 5: Figure S4. — Parallel TF detection by bead assay. Representative image of a parallel PCR readout (PA treated HepG2 samples). (DOCX 580 kb) [file 12896_2016_293_MOESM5_ESM.docx]
